# AP2/ERF Transcription Factor Regulatory Networks in Hormone and Abiotic Stress Responses in *Arabidopsis*

**DOI:** 10.3389/fpls.2019.00228

**Published:** 2019-02-28

**Authors:** Zhouli Xie, Trevor M. Nolan, Hao Jiang, Yanhai Yin

**Affiliations:** Department of Genetics, Development and Cell Biology and Plant Science Institute, Iowa State University, Ames, IA, United States

**Keywords:** AP2/ERF, plant stress, plant growth, plant hormones, gene regulatory network

## Abstract

Dynamic environmental changes such as extreme temperature, water scarcity and high salinity affect plant growth, survival, and reproduction. Plants have evolved sophisticated regulatory mechanisms to adapt to these unfavorable conditions, many of which interface with plant hormone signaling pathways. Abiotic stresses alter the production and distribution of phytohormones that in turn mediate stress responses at least in part through hormone- and stress-responsive transcription factors. Among these, the APETALA2/ETHYLENE RESPONSIVE FACTOR (AP2/ERF) family transcription factors (AP2/ERFs) have emerged as key regulators of various stress responses, in which they also respond to hormones with improved plant survival during stress conditions. Apart from participation in specific stresses, AP2/ERFs are involved in a wide range of stress tolerance, enabling them to form an interconnected stress regulatory network. Additionally, many AP2/ERFs respond to the plant hormones abscisic acid (ABA) and ethylene (ET) to help activate ABA and ET dependent and independent stress-responsive genes. While some AP2/ERFs are implicated in growth and developmental processes mediated by gibberellins (GAs), cytokinins (CTK), and brassinosteroids (BRs). The involvement of AP2/ERFs in hormone signaling adds the complexity of stress regulatory network. In this review, we summarize recent studies on AP2/ERF transcription factors in hormonal and abiotic stress responses with an emphasis on selected family members in *Arabidopsis*. In addition, we leverage publically available *Arabidopsis* gene networks and transcriptome data to investigate AP2/ERF regulatory networks, providing context and important clues about the roles of diverse AP2/ERFs in controlling hormone and stress responses.

## Introduction

Abiotic stresses such as water scarcity, extreme temperature and high salinity lead to arrested plant growth and ultimately result in massive agricultural losses (Fahad et al., 2017). Plants have developed sophisticated regulatory mechanisms to respond to external stress signals in a timely manner to ensure optimal growth and stress tolerance. Among these, hormonal signaling pathways and stress responsive transcriptional factors function together to form an interconnected network (Chen et al., 2017; Ye et al., 2017). In addition, environmental changes are often multifactorial, with several stresses occurring simultaneously. Instead of the linear stress signaling pathways, these regulatory components lead to more complex responses (Van den Broeck et al., 2017). Under abiotic stress conditions, stress hormones such as abscisic acids (ABA) and ethylene (ET) are induced, whereas the production and distribution of growth promoting hormones, such as gibberellins (GAs), brassinosteroids (BRs), and cytokinins (CTK) are also altered to enable optimal responses (Verma et al., 2016). The regulation of plant hormone signaling during abiotic stresses is partially mediated by hormone- and stress-responsive transcription factors (Nolan T. et al., 2017; Bechtold and Field, 2018).

APETALA2/ETHYLENE RESPONSIVE FACTOR (AP2/ERF) family transcription factors (AP2/ERFs) have emerged as key regulators of several abiotic stresses and respond to multiple hormones (Dietz et al., 2010; Mizoi et al., 2012; Chandler, 2018). Numerous *AP2/ERFs* mutants with altered abiotic stress responses and hormone sensitivity have been identified, positioning this family of transcription factors as promising candidates to study the interactions between abiotic stresses and hormones. Several properties of AP2/ERFs, such as induction upon specific stresses and diverse DNA binding preferences, enable these transcription factors to integrate responses of multiple stimuli and participate in different regulatory processes. In this review, we focus on *Arabidopsis thaliana* to summarize the regulation and function of AP2/ERFs in hormone and abiotic stress responses. The function of AP2/ERFs in crops has been extensively reviewed elsewhere (Abiri et al., 2017; Kulkarni et al., 2017; Phukan et al., 2017). Thus, only a few examples of AP2/ERFs in other species will be discussed. Finally, we investigate *AP2/ERFs* regulatory networks using publicly available transcriptome data to verify known and uncover novel roles of *AP2/ERFs* in hormone and stress responses.

## Overview of AP2/ERF Family Transcription Factors

AP2/ERFs are characterized by an APETALA2 (AP2)/Ethylene Responsive Element Binding Factor (EREB) domain, which consists of 40–70 conserved amino acids involved in DNA binding (Sakuma et al., 2002; Feng et al., 2005; Nakano et al., 2006). AP2/ERFs contain the four major subfamilies: APETALA2 (AP2), RELATED TO ABSCISIC ACID INSENSITIVE 3/VIVIPAROUS 1 (RAV), DEHYDRATION-RESPONSIVE ELEMENT BINDING proteins (DREBs) (subgroup A1–A6) and ETHYLENE RESPONSIVE FACTORS (ERFs) (subgroup V-X) (Sakuma et al., 2002; Nakano et al., 2006). As transcription factors, AP2/ERFs regulate genes involved in diverse biological processes such as growth, development, hormone and stress responses through several mechanisms including transcriptional and post-translational control (Dietz et al., 2010; Mizoi et al., 2012; Licausi et al., 2013; Gibbs et al., 2015; Chandler, 2018).

### Transcriptional Regulation of *AP2/ERFs*

**FIGURE 1:**
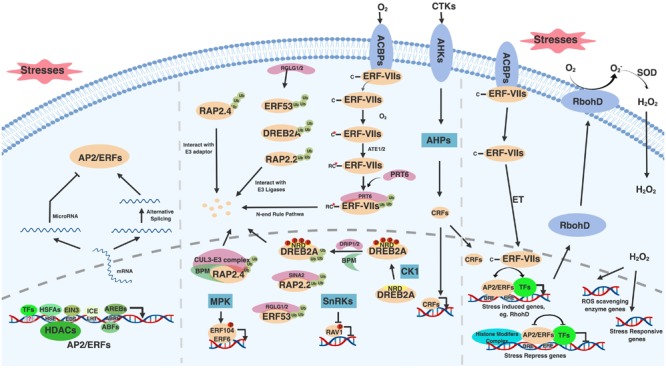
General regulatory mechanisms of AP2/ERF family transcription factors. AP2/ERFs are regulated by multiple stresses and stimuli at transcription, translation and protein modification levels. Upon stresses, *AP2/ERFs* are induced though *cis*-elements presented in their promoter regions (left bottom). These *cis*-elements include HSE, EBS, LRT, ABRE and many other unknown binding sites that respond to HSFAs, EIN3, ICE, AREBs and other transcription factors, respectively. The transcription of *AP2/ERFs* is also regulated by HDACs. Alternative splicing helps generate *AP2/ERFs* functional mRNA (left). Under normal condition, AP2/ERFs have adverse effect on plant growth and development and thus need to be eliminated. miroRNA mediated *AP2/ERF* silence is one of the ways to inhibit AP2/ERFs translation (left). E3 ubiquitin ligases involved proteasome degradation and phosphorylation mediated by kinases provide additional ways to regulate AP2/ERFs protein levels and activity (middle). These E3 ligases include DRIP1/2, RGLG1/2, SINA2, and PRT6, which mediate DREB2A, ERF53, RAP2.2, and ERF-VIIs degradation, respectively. ERF-VIIs undergo N-end rule mediated degradation under normal condition. BPM provides RAP2.4 docking adaptor for CUL3-E3 complex. MPKs and SnRKs mediated phosphorylation activates and represses ERF104 and RAV1 function, respectively. Under stresses, AP2/ERFs including CRFs and ERF-VIIs translocate into nucleus, bind to conserved or diverged DNA binding sites, interact with many other transcription factors and histone modification complex to either activate or repress stress responsive genes expression (right). Among stress induced genes, RbohD is responsive for ROS and H_2_O_2_ generation. Induced H_2_O_2_ serves as a signal messenger to active ROS scavenging enzyme genes and stress responsive genes expression. Figure is created with BioRender.

*AP2/ERFs* expression is tightly regulated to enable proper stress responses. Gene expression profiling studies have shown that most *AP2/ERFs* are expressed at low levels under normal conditions, whereas the expression can be induced or repressed at certain growth stages, by hormones and stress stimuli (Feng et al., 2005; Li et al., 2017a; Owji et al., 2017). In many cases, *AP2/ERFs* expression is regulated by the conserved *cis-*elements present in their promoter regions, or a combination of multiple responsive elements (Figure 1 left). For example, *Arabidopsis DREB2A* is highly induced under dehydration and heat conditions (Liu et al., 1998). The expression of *DREB2A* is controlled by HEAT SHOCK FACTOR1 (HSFA1) and ABRE-BINDING PROTEINs/ABRE-BINDING FACTOR 3 (AREB1/AREB2/ABF3) through binding to Heat Shock Element (HSE) and ABA Response Element (ABRE) motifs on its promoter, respectively (Kim et al., 2011; Liu et al., 2011). Moreover, by aligning 20 *Arabidopsis DREB* gene promoters to the motifs related to abiotic stresses, it was found that *DREB1A/CBF3, DREB1C/CBF2, DREB2C, DREB2G*, and *DEAR3* have the most types of these motifs including HSE motif to which heat shock factors bind, Low-Temperature Responsive element (LTR) that is important for the induction of cold regulated genes, and ABRE motif that responds to ABA (Sazegari et al., 2015). *AP2/ERFs* whose promoters contain ethylene-responsive (EBS) motifs are also likely to respond to ET signaling (Zhang et al., 2011).

Moreover, *AP2/ERFs* expression might be affected by histone modifications. Histone modifications such as phosphorylation, ubiquitination, methylation, and acetylation can either activate or repress transcription by creating more “open” or “closed” chromatin configurations (Pfluger and Wagner, 2007). A study in peanut (*Arachis hypogaea* L.) showed that the general inhibition of histone deacetylases (HDACs) and Polyethylene Glycol (PEG) treatment induced the acetylation around peanut *DREB1* (*AhDREB1*) promoter region and resulted in increased *AhDREB1* expression (Zhang B. et al., 2018). Given that chromatin features such as histone variants and post-translational histone modifications are altered by abiotic stresses, and these influences could be inherited to the next generation (Asensi-Fabado et al., 2017), the study of epigenetic regulation of AP2/ERFs will advance our understating the mechanisms controlling AP2/ERFs.

Another point of AP2/ERF regulation occurs at the level of splicing. Alternative splicing is required to create a functional isoform for some *AP2/ERFs* in rice (*Oryza sativa*) (*OsDREB2A/2B*) (Matsukura et al., 2010), maize (*Zea mays*) (*ZmDREB2A*) (Qin et al., 2007), wheat (*Triticum aestivum*) (*WDREB2*) (Egawa et al., 2006), and barley (*Hordeum vulgare*) (*HvDRF1*) (Xue and Loveridge, 2004). In these cases, plants produce an inactive *AP2/ERF* form containing stop codons before the DNA binding domain during normal conditions, while under stress conditions, the exon with a premature stop codons is excluded to generate a functional transcription factor. In addition, microRNA (miRNA)-mediated RNA silencing and translation repression was shown to regulate *AP2/ERFs*. miRNA172 targets *Arabidopsis AP2* messenger RNA to inhibit its translation (Chen, 2004). Several other miRNAs such as miRNA156 and miRNA838 have also been shown to regulate *AP2/ERFs* in common bean (*Phaseolus vulgaris*) (Kavas et al., 2015).

### Post-translational Modifications of AP2/ERFs

In addition to transcriptional control, post-transcriptional regulation modulates the activity and abundance of AP2/ERF proteins (Figure 1 middle). One of the modifications, phosphorylation, affects AP2/ERF protein transactivity and stability. In *Arabidopsis*, SNF1-RELATED PROTEIN KINASES (SnRKs), a positive regulator in ABA signaling pathway, interacts with and phosphorylates RAV1 to inhibit its transcriptional repression function (Feng et al., 2014), while ERF104 and ERF6 are phosphorylated by mitogen-activated protein kinases (MPKs) to positively regulate pathogen responses (Bethke et al., 2009; Meng et al., 2013). The stability of *Arabidopsis* DREB2A is also affected by phosphorylation. DREB2A is destabilized by phosphorylation on its negative regulatory domain (NRD), which is probably mediated by Casein Kinase 1 (CK1) (Mizoi et al., 2018). Indeed, many kinases such as GSK3-like kinase BR-INSENSITIVE 2 (BIN2) and SnRKs have been reported to mediate abiotic stresses (Nolan T. et al., 2017). However, information about how these kinases regulate AP2/ERFs is limited. Therefore, future studies such as phosphoproteomics under abiotic stresses and upon perturbation of specific kinases could help identify AP2/ERF protein modifications, leading to potential strategies to modify the protein activity and stability of AP2/ERFs to confer abiotic stress tolerance.

The protein stability of AP2/ERFs is also regulated by ubiquitin-mediated protein degradation through the 26S proteasome pathway as revealed in *Arabidopsis* (Figure 1 middle). Under non-stress conditions, DREB2A, ERF53, and ERF75/RAP2.2 proteins are directly ubiquitinated by RING domain E3 ligases DREB2A-INTERACTING PROTEIN1/2 (DRIP1/2), RAlGDS-LIKE (RGLG1/2), and SEVEN IN ABSENTIA OF ARABIDOPSIS2 (SINAT2), respectively (Qin et al., 2008; Cheng et al., 2012; Papdi et al., 2015). A CUL3-based E3 ligase adaptor BTB/POZ AND MATH DOMAIN proteins (BPMs) also mediates DREB2A and ERF59/RAP2.4 degradation (Weber and Hellmann, 2009; Morimoto et al., 2017). Additionally, the stability of several *Arabidopsis* ERF-VIIs is controlled by the N-end rule pathway, where the N terminal Met (Nt-Met) of ERF71/HRE2, ERF72/RAP2.3, and ERF74/RAP2.12 is removed and the second amino acid Cys (Nt-Cys) is oxidized into cysteine sulfinic/sulfonic acid in an oxygen-dependent manner (Gibbs et al., 2015). This process triggers ubiquitination mediated by the E3 ligase PROTEOLYSIS 6 (PRT6) (Gibbs et al., 2011; Licausi et al., 2011; Abbas et al., 2015). Particularly, RAP2.12 interactes with acyl-CoA binding protein 1/2 (ACBP1/2) and is localized on the plasma membrane under normal conditions. During the limited oxygen conditions, RAP2.12 de-associates from ACBP1/2 and moves into the nucleus by an unknown mechanism (Gibbs et al., 2015). ET also promotes RAP2.3 nuclear localization (Kim et al., 2018). Overall, a number of components have been identified that regulate AP2/ERF stability, localization and activity. Since many AP2/ERFs are involved in stress responses, this tight regulation of AP2/ERF protein likely ensures that plants effectively respond to environmental stimuli without ectopic activation of AP2/ERF-mediated stress responses.

### DNA Binding Diversity of AP2/ERFs

To regulate target genes, AP2/ERFs have conserved DNA binding preferences (Nakano et al., 2006). Typically, DREBs recognize Dehydration-Responsive or C-Repeat Element (DRE/CRT) with A/GCCGAC core sequence on stress-responsive genes to confer resistance to drought, cold and heat abiotic stresses. ERFs bind to Ethylene-Response Element (ERE) with AGCCGCC core sequence (also known as GCC-box) to confer resistance to biotic stresses (Shinozaki and Yamaguchi-Shinozaki, 2000; Guo and Ecker, 2004; Franco-Zorrilla et al., 2014). However, many *Arabidpsis* DREBs and ERFs have been reported to bind to both DRE/CRT and ERE elements, implying their potential roles in both abiotic and biotic stress. For instance, DREBs (including TINY, CBF1, ERF53, RAP2.4, and TG/RAP2.4A) and ERFs (including ERF1, ERF4, and ERF71) bind to both DRE and ERE elements (Lin et al., 2008; Sun et al., 2008; Yang et al., 2009; Cheng et al., 2012; Zhu et al., 2014; Lee et al., 2015). Similarly, the conserved DNA binding preferences of AP2/ERF are also expanded to other species such as rice (Wan et al., 2011), wheat (Gao et al., 2018), maize (Liu et al., 2013), soybean (*Glycine max*) (Zhang et al., 2009), and tobacco (*Nicotiana tabacum*) (Park et al., 2001). Recently, the combination of high-throughput protein-binding microarray and relevant transcriptome data demonstrated that transcription factors with high structure identity share similar DNA binding sites, which also enables them to share some biological relevance and explains their functional redundancy (Franco-Zorrilla et al., 2014). It provides a way to predict unknown AP2/ERFs function. Additionally, AP2/ERFs recognize *cis*-elements that diverge significantly from these motifs (Table 1). For example, Coupling Element 1 (CE1: TGCCACCG), Coupling Element 3-like (CE3-like: CGCG), Hypoxia-Responsive Promoter Element (HRPE) and non-specific sequences are recognized by AP2/ERFs (Kagaya et al., 1999; Welsch et al., 2007; Shaikhali et al., 2008; Bossi et al., 2009; Zhu et al., 2010; Dinh et al., 2012; Chen et al., 2016; Gasch et al., 2016; Park et al., 2016). Through different partners upon binding to different sites, the diversity of AP2/ERFs DNA binding broadens the scope of target genes and might enable them to participate in different regulatory processes. To date, both *in vivo* (ChIP-chip and ChIP-seq) and *in vitro* (DAP-seq, SELEX-seq and Protein-Binding Microarrays (PBMs)) techniques are helpful to decipher the AP2/ERFs transcriptional regulatory code (Franco-Zorrilla et al., 2014; O’Malley et al., 2016; Bartlett et al., 2017).

**Table 1:** The diverse DNA binding preference of AP2/ERFs in Arabidopsis.

Phylogenetic classification	Gene name	Interacting *cis*-element	Regulation traits	References
A1	CBF1	DRE/CRT and ERE	Positively regulate cold	Yang et al., 2009
A3	ABI4	CE1	Activator and repressor to mediate ABA signaling pathway	Bossi et al., 2009
A4	TINY	DRE/CRT and ERE	Up-regulate by ABA and ET as well as abiotic stresses, but negatively regulate plant development	Sun et al., 2008
A5	ORA47	(NC/GT)CGNCCA	Negatively regulate ABA signaling; mediate cross talk between JA and ABA	Chen et al., 2016
A6	ERF53	DRE/CRT & ERE	Positively regulate drought	Cheng et al., 2012
	RAP2.4	DRE/CRT and ERE	Positively regulate drought but negatively regulate plant development	Lin et al., 2008
	RAP2.4A	DRE/CRT and ERE and CE3-like	Positively regulate drought	Shaikhali et al., 2008; Zhu et al., 2014
ERF-VII	ERF71/HRE2	DRE/CRT and ERE	Positively regulate flooding tolerance and root cell expansion	Lee et al., 2015
	ERF72/RAP2.3	HRPE	Positively regulate hypoxia tolerance	Gasch et al., 2016
	ERF74/RAP2.12	HRPE		Gasch et al., 2016
	ERF75/RAP2.2	HRPE and ATCTA		Welsch et al., 2007; Gasch et al., 2016
ERF-VIII	ERF4	DRE/CRT and ERE	Negatively regulate ET and ABA response, as well as iron deficiency	Yang et al., 2005, 2009; Liu et al., 2017
ERF-IX	ERF1	DRE/CRT and ERE	Positively regulate salt, drought, and heat stresses	Yang et al., 2009; Cheng et al., 2013
ERF-X	RAP2.6	ERE and CE1	Positively regulate ABA and abiotic stresses but negative regulate plant development	Zhu et al., 2010
AP2	AP2	T/A-rich	Positively regulate floral organ identity	Dinh et al., 2012
	WRI4	CAACA, CAA/CA/CTG, CATGCA, and ATCGAG elements	Positively regulate cuticular wax biosynthesis	Park et al., 2016
RAV	RAV	CAACA and CACCTG	Negatively regulate ABA signaling during seed germination and positively regulate leaf senescence	Kagaya et al., 1999; Woo et al., 2010; Feng et al., 2014

### Transcriptional Regulation of Target Genes by AP2/ERFs

Through directly binding to target gene promoters, AP2/ERFs can either activate or repress target gene expression (Figure 1 right). Besides an N-terminal DNA binding domain, the C-terminal activation domain of AP2/ERFs mediates the activation of target gene expression in *Arabidopsis* and rice (Nakano et al., 2006). Recently, an activation EDLL motif was identified from *Arabidopsis* ERF98 and AP2 subfamily. The EDLL motif is strong enough to override the repression effect mediated by ERF-associated amphiphilic repression (EAR) motif, which emphasizes the transactivation activity of AP2/ERFs (Tiwari et al., 2012). However, AP2/ERFs containing an EAR motif with the consensus sequence LxLxL or DLNxxP, or B3 repression domain (BRD) with R/KLFGV sequence exhibit a repressive effect on target genes (Ikeda and Ohme-Takagi, 2009; Kagale and Rozwadowski, 2011). The EAR motif containing AP2/ERFs also interact and recruit transcription co-repressors like TOPLESS (TPL) and TOPLESS-RELATED (TPR) (Causier et al., 2012) or histone modifiers to suppress target gene expression (Song et al., 2005; Song and Galbraith, 2006). In *Arabidopsis*, ERF7 interacts with a human global co-repressor SIN3 homolog (ATSIN3) that in turn interacts with Histone Deacetylase 19 (HDA19) (Song et al., 2005). Similarly, ERF3 interacts with SIN3 Associated Polypeptide P18 (SAP18) and then recruits HDA19 to repress gene expression (Song and Galbraith, 2006). The BRD motif containing *Arabidopsis* RAV1 and RAV2 also display repressive activities (Ikeda and Ohme-Takagi, 2009). There is a report demonstrating that genes co-regulated with corresponding AP2/ERFs were enriched with the AP2/ERFs targets. Therefore, by analyzing the enrichment of cognate motifs in AP2/ERFs co-regulated genes, it is possible to identify putative target genes of transcription factors and predict their biological functions (Franco-Zorrilla et al., 2014).

## AP2/ERF Regulatory Networks in Abiotic Stresses

AP2/ERFs regulate numerous abiotic stresses such as cold, drought, heat, salt, and freezing (Lata and Prasad, 2011; Mizoi et al., 2012; Licausi et al., 2013; Phukan et al., 2017). Although many AP2/ERFs are proposed to form an abiotic stress-specific regulatory network, the ability of AP2/ERFs to respond to multiple stimuli and regulate different stresses enable them to form a more complex stress response network. In this network, AP2/ERFs also respond to abiotic stresses with varying dynamic patterns: some AP2/ERFs are induced quickly and continuously, whereas others are regulated by prolonged stress, which indicates they might have mutual influence on each other’s function (Van den Broeck et al., 2017). However, the detailed mechanisms of how different AP2/ERFs cooperate or antagonize with each other are yet to be established. Therefore, in addition to studying the function of individual transcription factors in this family, it is also necessary to study the relationship between different AP2/ERFs in abiotic stress responses. Here we summarize recent works on the DREBs and ERFs regulation in cold, drought, heat and salt stress responses particularly in *Arabidopsis*, and we also discuss the mutual regulation of different AP2/ERFs.

### DREBs: Major Regulators in Cold, Drought, Heat, and Salt Stress Responses

**FIGURE 2:**
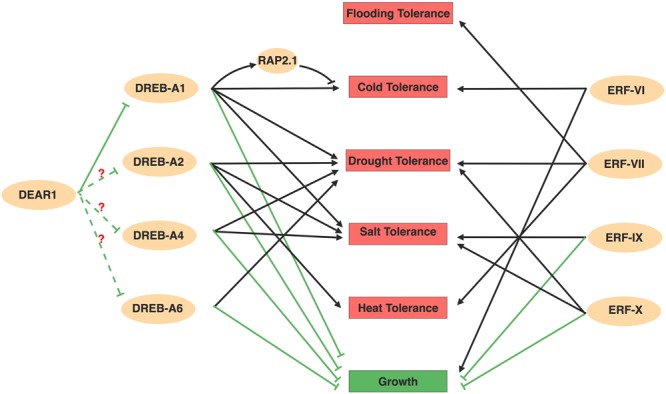
Overview about AP2/ERFs mediated abiotic stresses. Members in DREBs and ERFs subfamily positively regulate numerous abiotic stresses, but negatively regulate plant growth. The ability to regulate several stresses simultaneously form a comprehensive regulatory network. Among the network, DREB-A1 family might act as repressors at the upstream of DREBs with question mark. Arrows and bar ends indicate activation and repression effect, respectively. Figure is created with BioRender.

DREBs have been extensively examined in abiotic stresses, where they respond to and positively regulate cold, drought, heat and salt tolerance by directly regulating stress-responsive genes (Figure 2). Among these, DREB1s (DREB-A1 subgroup) containing several C-Repeat-Binding Factors (CBFs) play major roles in acquisition of freezing tolerance (Chinnusamy et al., 2003). CBFs together with another major cold responsive transcription factor, Inducer of CBF Expression (ICE), establish a central cold response pathway to activate a majority of DRE containing Cold Responsive Genes (*CORs*) in *Arabidopsis* (Zhao et al., 2016; Liu J. et al., 2018). *CORs* encode Late Embryogenesis Abundant (LEA) proteins and enzymes for sugar metabolism and fatty acid desaturation that provide the protection for plants from cold stress (Maruyama et al., 2009). The roles of *Arabidopsis* CBFs in stress responses have been characterized by genetics using *cbf* mutants generated by CRISPR/Cas9 genome editing techniques and *CBFs* overexpression lines, as well as transcriptome analysis (Park et al., 2015; Zhao et al., 2016). CBFs are also reported to positively regulate plant drought and salt tolerance, which might due to a common set of stress responsive genes (Kasuga et al., 1999; Zhao and Zhu, 2016). Despite having a clear function in cold response pathways, how CBFs regulate different stresses and the mechanisms by which they confer stress tolerance are still unknown. Environmental changes are usually multifactorial and several stresses often occur simultaneously. Therefore, the multiple roles of CBFs in abiotic stresses might be necessary for plants to overcome stresses and it will be interesting to examine how CBFs regulate genes under different stress conditions.

In addition to CBFs, transcriptome profiling in *Arabidopsis* also identified several other regulators that are activated during cold acclimation, including members in the DREB-A5 group (Fowler and Thomashow, 2002). The DREB-A5 group contains six members with EAR motifs (DEARs) acting as transcriptional repressors on DRE motif containing genes (Nakano et al., 2006), which provides a repression effect in the DREB-regulated abiotic stress network. Among these, DEAR1 likely acts upstream of CBFs, while DEAR6/RAP2.1 acts downstream of CBFs (Tsutsui et al., 2009; Dong and Liu, 2010). Overexpressed *DEAR1* suppressed the cold induced expression of *CBFs* and displayed reduced plant freezing tolerance (Tsutsui et al., 2009). RAP2.1 is induced by cold, as well as in plants constitutively overexpressing *CBFs*, but the induction of RAP2.1 by cold has a later onset than the induction of *CBF2* (Dong and Liu, 2010). RAP2.1 was first identified as a downstream CBFs subregulon and negatively modifies plants cold tolerance (Fowler and Thomashow, 2002). The presence of the DRE motif in the RAP2.1 promoter region also suggests that *RAP2.1* might be a direct target of CBFs (Dong and Liu, 2010). Although the integration of DEAR1 and RAP2.1 in the CBF pathway still needs to be examined using genetics, this negative regulation mechanism might provide checks and balances that minimize the adverse effects of prolonged stress responses. In addition to the negative role of RAP2.1 in cold stress, it also negatively regulates drought tolerance, in that overexpression of *RAP2.1* resulted in sensitivity to drought (Dong and Liu, 2010). However, how RAP2.1 regulates drought response, whether it regulates other abiotic stresses and what other DEARs function in abiotic stresses are interesting questions to answer in the future. The checks and balances of DREBs’ function in stress were also revealed in *Brassica napus*, where two groups DREBs (Group I and Group II) regulate cold stress responsive genes sequentially. The early induced Group I DREBs activate cold stress response pathways, whereas the Group II DREBs which are expressed later competitively inhibited Group I DREBs function (Zhao et al., 2006).

DREB2s from DREB-A2 are mostly involved in plant drought and heat tolerance, which has been reviewed in detail elsewhere (Mizoi et al., 2012). Briefly, DREB2s are induced upon drought and heat, and positively regulate DRE containing drought responsive genes such as *LEAs*, and heat responsive genes such as heat chaperons (Maruyama et al., 2009). Moreover, members in DREB-A4 family such as HARDY (HRD) and in DREB-A6 family such as ERF53, RAP2.4, and TG/RAP2.4A also positively regulate drought and salt tolerance (Karaba et al., 2007; Lin et al., 2008; Cheng et al., 2012; Zhu et al., 2014). Overexpression of *HRD* in *Arabidopsis* or rice remarkably improved plant drought and salt tolerance (Karaba et al., 2007). Overexpression of *TRANSLUCENT GREEN* (*TG*) resulted in vitrified leaves with increased water content in cells, leading to increased drought tolerance (Zhu et al., 2014). TG regulates cell water homeostasis mainly by directly activating several aquaporin genes (Rae et al., 2011; Zhu et al., 2014), as well as Ascorbate Peroxidases (*APx*) genes encoding chloroplast peroxidases that functions to protect against photo-oxidative stresses caused by Reactive Oxygen Species (ROS) (Rudnik et al., 2017).

Beside *Arabidopsis*, DREBs’ improvement in stress tolerance has been extensively studied in various crops such as OsDREB1s and OsDREB2s in rice (Dubouzet et al., 2003; Matsukura et al., 2010), ZmDREB2A in maize (Qin et al., 2007), TaDREB1 in wheat (Shen et al., 2003), and HvDRF1 in barley (Xue and Loveridge, 2004). These DREBs provide abundant candidate genes for the engineering of stress tolerance in crops. However, given that DREBs are a major family of AP2/ERF transcription factors that integrate multiple abiotic stress signals, cooperate or antagonize one another, and modulate downstream stress responsive genes, studying the DREB gene regulatory network will provide a platform for a more comprehensive understanding of abiotic stress responses and guide the genetic engineering of crops.

### ERF, AP2, and RAV Subfamily Members in Freezing, Hypoxia, and Salt Stress Responses

Members in ERF subfamily also contribute to abiotic stress responses (Licausi et al., 2013). Recently, two groups of ERFs have emerged as central players of abiotic stress regulation in *Arabidopsis* (Figure 2). *CYTOKININ RESPONSE FACTORS* (*CRFs*) in ERF-VI subfamily are induced by CTK as well as multiple abiotic stresses to positively regulate osmotic and freezing tolerance (Rashotte et al., 2006; Rashotte and Goertzen, 2010). *CRF6*, whose induction is dependent on the perception of CTK, alleviated the H_2_O_2_ damage on plants to positively regulate oxidative response (Zwack et al., 2016b). CRF4, one of several CRFs not transcriptionally regulated by CTK, positively regulates freezing tolerance by promoting *CORs* expression (Zwack et al., 2016a). However, the mechanisms by which CRFs confer stress tolerance remain to be determined.

Members of ERF-VII subfamily in *Arabidopsis* as well as rice have been demonstrated to play major roles in flooding, low oxygen (hypoxia) and submergence tolerance and their redundant function in hypoxia responses has been reviewed (Bailey-Serres et al., 2012; Bui et al., 2015; Gibbs et al., 2015). For example, rice SUBMERGENCE 1A (SUB1A), and SNORKEL1/2 (SK1/SK2) positively regulate flooding tolerance by two opposite mechanisms: SUB1A mediates a quiescence strategy associated with reduced growth and respiration whereas SK1/SK2 promote a deep-water escape strategy allowing rapid growth of petioles, stems, and vascular changes (Hattori et al., 2009; Locke et al., 2018).

In *Arabidopsis*, ERF-VIIs have conserved N-terminal domains that allow them to be degraded under anoxia conditions though oxygen-dependent N-end rule pathway (Gibbs et al., 2015). Five members including *ERF71/HRE2, ERF72/RAP2.3, ERF73/HRE1, ERF74/RAP2.12*, and *ERF75/ RAP2.2* are induced by limited oxygen. With limited oxygen, these ERF-VIIs accumulate and positively regulate hypoxia responsive genes involved in sugar metabolism, fermentation and ET biosynthesis to achieve hypoxia tolerance. Apart from hypoxia responses, ERF-VIIs also regulate oxidative and osmotic stresses. Overexpression of *RAP2.2, RAP2.3* and *RAP2.12* (RAPs) results in a higher survival rate from low oxygen, oxidative and osmotic stresses, while *rap2.12-2 rap2.3-1* double mutants are sensitive to these stresses (Papdi et al., 2015; Yao et al., 2017b).

RESPIRATORY BURST OXIDASE HOMOLOG D (RbohD), a NADPH oxidase, helps to generate ROS burst (Yao et al., 2017b). It is reported that RAPs regulate abiotic stresses via an RbohD-dependent mechanism. Apart from being a toxic by-product of biochemical processes, ROS serve as signaling molecules to trigger stress responses and transduce signals crossing cells according to its lower molecular weight and fast cell diffusion (Qi et al., 2018). First it was found that the ROS production and RbohD expression were compromised in single *erf7*4 and double *erf74 erf75* mutants at an early stage, which resulted in compromised stress responsive gene expression and stress tolerance. Given that ERF74 binds to *RbohD* promoter to activate its expression, the RbohD dependent ROS activation was essential for ERF74 and ERF75 mediated hypoxia resistance. However, too much ROS can cause cell injury and cell death. To overcome adverse effects of ROS, overexpression of *ERF74* promoted increased ROS scavenging enzymes and stress responsive genes at later stage. Therefore, ERF74 acts as an on-off switch to control RbohD-dependent ROS burst in response to different stresses in *Arabidopsis* (Yao et al., 2017b). This newly identified mechanism provides more details and divides the stress response into early and later stages, as well as ROS balance. One example that rice ERFs OsLG3 induced ROS scavenging to positively regulate stress tolerance was reported recently, suggesting the similar mechanism exists in rice (Xiong et al., 2018).

Additionally, many other *Arabidopsis* ERFs also regulate abiotic stresses. ERF1 and Ethylene- and Salt-inducible ERF genes (ESEs) in ERF-IX group positively regulate plant salinity tolerance by promoting salt responsive gene expression (Zhang et al., 2011). ERF6, another member in ERF-IX group, triggers growth inhibition to confer long-term osmotic stress tolerance (Dubois et al., 2013). RAP2.6L from ERF-X subgroup improves drought and salt tolerance (Yang et al., 2009; Liu et al., 2012). Additionally, Arabidopsis RAVs, especially AP2s, play central roles in developmental processes, such as organ number and size control, shoot and root meristem maintenance, flower initiation and growth (Osnato et al., 2012; Horstman et al., 2014). Members in these subfamilies are also reported to mediate diverse abiotic stress responses. AINTEGUMENTA (ANT) controls organ cell number and size throughout shoot development. ANT also negatively regulates salt tolerance by repressing SOS3-LIKE CALCIUM BINDING PROTEIN 8 (*SCABP8/CBL10*), a putative Ca^2+^ sensor that protects *Arabidopsis* shoots against salt stress and maintains ion homeostasis (Meng et al., 2015a). Overexpression of *Arabidopsis* RAV1 and RAV2 in cotton increased fiber length and even obtained the same yield under drought stress compared with control conditions (Mittal et al., 2015).

Similar as DREBs, ERFs in other plant species like rice, wheat and tomato are also involved in a broad range of abiotic stresses (Abiri et al., 2017; Phukan et al., 2017). Overall, these findings provide the potential of engineering high-efficiency crops under stress conditions. In summary, ERFs function to receive multiple stress signals and control a diverse set of stress responsive genes, where many ERFs have cooperative or antagonistic regulation on stress responses. Therefore, constructing ERF-specific gene regulatory networks would be interesting to provide insight as to how ERFs function as a unit to regulate common downstream genes.

## Integration of AP2/ERFs With Hormone Responses

**FIGURE 3:**
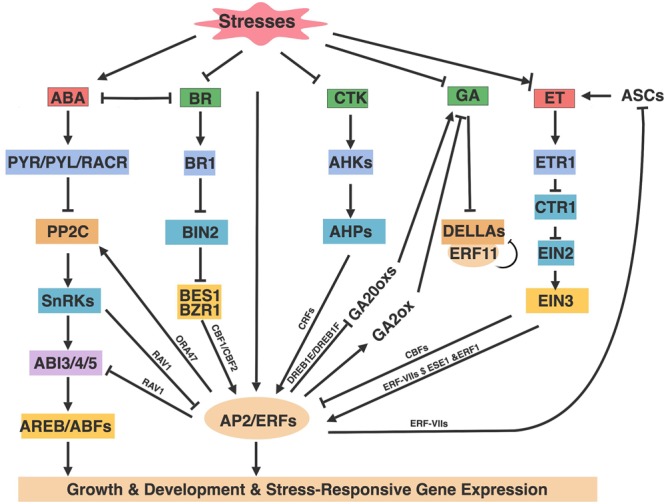
AP2/ERFs roles in hormone pathways. Abiotic stresses alter the production and distribution of phytohormones that in turn mediate stresses responses through hormone signaling components and AP2/ERFs. Arrows and bar ends indicate activation and repression effect, respectively. Figure is created with BioRender.

In addition to directly regulating abiotic stresses, AP2/ERFs are also involved in hormone signaling and hormone mediated-stress responses. Plant hormones affect abiotic stresses by triggering a wide range of physiological processes (Kazan, 2013, 2015; Colebrook et al., 2014; Khan et al., 2015; Muller and Munne-Bosch, 2015; Tao et al., 2015; Sah et al., 2016; Nolan T. et al., 2017). ABA and ET are major stress hormones that are induced under abiotic stress conditions and regulate stress responses associated with AP2/ERFs (Kazan, 2015; Sah et al., 2016). GAs, CTK, and BRs are growth-related hormones that promote cell growth, proliferation and differentiation. It is becoming increasingly evident that these growth-related hormones also have direct and/or indirect effects on abiotic stresses (Kazan, 2013; Colebrook et al., 2014; Nolan T. et al., 2017). Because stress tolerance is often associated with trade-offs between growth and stress, abiotic stress-tolerant plants usually have lower growth rates and productivity (Bechtold and Field, 2018). Numerous studies in *Arabidopsis* have shown AP2/ERFs confer plant stresses tolerance associated with growth losses (Figure 2), such as CBFs, DREB2A, HARDY, TG, ERF6, and RAP2.6 (Kasuga et al., 1999; Sakuma et al., 2006; Karaba et al., 2007; Sharabi-Schwager et al., 2010; Krishnaswamy et al., 2011; Dubois et al., 2013; Zhu et al., 2014). However, the growth loss can be minimized by driving expression via the stress-induced *RD29A* promoter (Kasuga et al., 1999; Sakuma et al., 2006; Matsukura et al., 2010). Thus, in addition to understanding the basis of AP2/ERFs in abiotic stresses, it is also important to explore their roles in the hormone-regulated stresses responses. AP2/ERFs are involved in plant hormone-mediated abiotic stresses through the following aspects: (1) *AP2/ERFs* transcripts are regulated by hormones; (2) AP2/ERFs alter hormone sensitivity and gene expression by cooperating or antagonizing various hormone signaling components; (3) AP2/ERFs regulate hormone biosynthesis or metabolism via feedback regulation (Figure 3). In the following sections we emphasize how abiotic stress-induced (ABA and ET) and growth-promoting (GAs, CTK, and BRs) hormones are regulated by AP2/ERFs in *Arabidopsis* through these mechanisms.

### AP2/ERFs in ABA-Mediated Stress-Response

The plant hormone ABA is a pivotal hormone that regulates abiotic stress responses including drought, salinity, cold and heat stresses. ABA exerts a protective function through inducing stomata closure, modulating root architecture, and promoting the synthesis of osmolytes (Cutler et al., 2010; Sah et al., 2016). During stress conditions such as water deprivation and osmotic pressure, the rate-limiting ABA biosynthetic enzyme Nine-*cis*-Epoxycarotenoid Dioxygenase (*NCED*) is rapidly up-regulated to promote ABA biosynthesis. ABA is then sensed by a large family of PYRABACTIN RESISTANCE1 /PYR1-LIKE/REGULATORY COMPONENTS OF ABA RECEPTORS (PYR/PLY/RCAR) receptors that interact with Protein Phosphatase 2C (PP2Cs) as a ternary complex to release PP2Cs’ inhibition on SnRK2 kinases (SnRK2s). The active SnRK2s phosphorylate downstream substrate proteins, including AREBs/ABFs, ion channels, and enzymes such as NADPH oxidases, thereby inducing ABA responses (Finkelstein, 2013; Sah et al., 2016). Generally, transcription factors regulate abiotic stresses through ABA-dependent or ABA-independent pathways.

A great deal of studies have shown that DREBs regulate ABA-independent abiotic stresses by directly binding to DRE/CRT motifs on stress responsive genes (Gilmour et al., 2004; Matsukura et al., 2010; Lata and Prasad, 2011; Mizoi et al., 2012; Zhu et al., 2014). However, AP2/ERFs are indispensable for ABA-dependent stress responses as well. *ANT* (Meng et al., 2015b), *ERF53* (Hsieh et al., 2013), *ERF-VIIs* (Papdi et al., 2015; Yao et al., 2017a), *RAP2.6L* (Liu et al., 2012), and *RAP2.6* (Zhu et al., 2010) in *Arabidopsis* are induced by ABA to up-regulate DRE- and ABRE- element containing genes. Rice OsERF71 positively regulates ABA signaling to alter root architecture and confer drought tolerance (Lee et al., 2017; Li et al., 2018). The combination of abiotic stresses and ABA also led to the further activation of *DREB2s* and stress inducible genes (Lee et al., 2016). ABA INSENSITIVE *4* (ABI4), a unique one in the DREB-A3 group is a key component of the ABA signaling pathway. Upon ABA and ROS accumulation under stress conditions, ABI4 represses CCAAT Binding Factor A (CBFA) (Zhang et al., 2013). CBFA is a subunit of the trimeric transcription complex of Heme Activator Proteins (HAPs). Repression of CBFA then allows other transcription factors to enter the transcription complex and improves the efficiency of stress responsive gene transcription (Zhang et al., 2013).

Apart from the positive effect of AP2/ERFs in ABA-mediated stress responses, many *Arabidopsis* studies have shown that AP2/ERFs also interrupt ABA signaling, resulting in reduced sensitivity on root growth inhibition and stomata closure (Figure 3). ERF18/ ORA47 activated the PP2C family phosphatase gene *ABI2*. At the same time ABI1 acted upstream of ORA47 to activate ORA47, leading to an ABI1-ORA47-ABI2 regulation loop that inhibits ABA signaling as well as drought tolerance (Chen et al., 2016). RAV1 inhibites ABA sensitivity on root growth by repressing *ABI3, ABI4* and *ABI5* expression (Feng et al., 2014). SnRK2.2, SnRK2.3, SnRK2.6 also interact with and phosphorylate RAV1 to inhibit RAV1’s transcriptional repression of targets genes (Feng et al., 2014).

### AP2/ERFs in Ethylene-Mediated Stress-Response

ET is also reported to regulate abiotic stress responses including salt, cold, and flooding (Kazan, 2015). ET is synthesized from the rate limiting enzymes ACC Synthases (ACSs), a major target for the regulation for ET production under stresses (Tao et al., 2015). The binding of ET with its receptor ETHYLENE INSENSITIVE 1 (ETR1) deactivates CONSTITUTIVE TRIPLE RESPONSE 1 (CTR1) kinase activity to release CTR1’s inhibition on ETHYLENE INSENSITIVE 2 (EIN2). Then the C-terminal of EIN2 translocates into the nucleus to activate *ETHYLENE INSENSITIVE 3* (*EIN3*) as well as the transcriptional cascade of ethylene-regulated genes (Qiao et al., 2012; Muller and Munne-Bosch, 2015).

In stress responses, AP2/ERFs, especially the ERF subfamily, are the major downstream regulatory factors of the ET signaling pathway (Licausi et al., 2013; Gibbs et al., 2015; Kazan, 2015; Muller and Munne-Bosch, 2015). ET regulates several aspects of *Arabidopsis* abiotic stress responses, including inhibiting *CBFs* to negatively regulate cold stress (Figure 3). Conversely, ET positively regulates flooding and submergence mediated by ERF-VIIs, and improves salt tolerance by activating *ERF1* and *ESEs*.

For ET-regulated cold response, the production of ET is inhibited after exposure to cold, which results in compromised cold tolerance (Shi et al., 2012). Consistently, ET insensitive mutants *etr1-1, ein2-5*, and *ein3-1* displayed increased freezing tolerance. EIN3 inhibits the expression of *CBFs* by directly binding to their promoters. In addition, ET plays crucial roles on plant survival and recovery from flooding, especially in rice. Flooding causes oxygen deficiency, which promotes ET production (Yang et al., 2011) and activates the expression of a set of *ERF-VIIs*, whose function in hypoxia is discussed above. ET also promotes RAP2.3 nuclear localization and advances ORA59 mediated ethylene responses which is dependent on RAP2.3 (Kim et al., 2018). However, ERF-VIIs regulate hypoxia response partially through ET-independent pathways. The induction of *ERF73/HRE1* under hypoxia was not completely abolished in ethylene-insensitive mutants or in the presence of ethylene biosynthesis inhibitors. ERF-VIIs also negatively regulate ET signaling and homeostasis probably via feedback regulations (Hinz et al., 2010; Yang et al., 2011). For instance, *HRE1-RNAi* seedlings displayed exaggerated triple responses; *ACSs* was decreased in *RAP2.2* overexpression plants, but up-regulated in *rap2.2-2* knockout mutant (Hinz et al., 2010). However, how ERF-VIIs control ET homeostasis via negative feedback mechanism under stresses needs further investigation.

ET also has complex regulation in salt stress, which has been extensively discussed (Kazan, 2015; Muller and Munne-Bosch, 2015; Tao et al., 2015). In *Arabidopsis*, ET signaling is required for plant tolerance to salinity stress as EIN3 activates *ERF1* and *ESEs* to activate downstream stress-related genes and promote salinity tolerance. However, knockout mutants of *ACSs* also led to salt tolerance, leading to an opposite conclusion in terms of ET signaling and salt tolerance. These different conclusions might be due to the different mutants, growth conditions or experimental setups used. Future investigation is necessary to further our understanding of the role of ET in plant salinity response.

### AP2/ERFs in GA-Mediated Stress-Response

The plant hormone GAs is known to promote plant growth and development. GAs have also been shown to regulate abiotic stresses, as reduced GA content slows down plant growth upon exposure to several abiotic stresses including cold, salt, and osmotic stresses (Claeys et al., 2012; Colebrook et al., 2014). GAs are synthesized through several key oxidases including GA 20-oxidases (GA20oxs) and GA 3-oxidases (GA3oxs), and catabolized by GA 2-oxidase (GA2ox) that depletes pools of GA precursors to maintain GA homeostasis (Phillips et al., 1995; Rieu et al., 2008). In the absence of GAs, a group of DELLA proteins (DELLAs) inhibit GA response, and this inhibition can be released by the degradation of DELLAs in the presence of GAs (Claeys et al., 2012). Generally, abiotic stresses cause reduction of GA content and signaling through the inhibition of GA biosynthesis enzymes mediated by *Arabidopsis* AP2/ERFs (Figure 3). DREB1E and DREB1F confer salt stress-induced growth retardation mostly through the repression of GA20oxs (Magome et al., 2004). *CBF1* and *ERF6* overexpression plants were sensitive to stress-induced growth retardation because of increased GA2oxs expression as well as the accumulation of DELLAs (Achard et al., 2008; Dubois et al., 2013). Conversely, ERF11 promotes plant internode elongation by activating GA biosynthesis, and expression of *GA3ox1* and *GA20oxs* are increased in *ERF11* overexpression plants (Zhou et al., 2016). Nevertheless, ERF11 and ERF6 show antagonistic regulation on stress-induced growth inhibition. ERF11 suppresses the extreme dwarf phenotype of *ERF6* overexpression plants and represses ERF6-induced gene expression (Dubois et al., 2015). The opposite regulation by ERF6 and ERF11 reveals that dynamic mechanisms must exist in plants to fine-tune and maintain the balance between plant growth and stress responses.

In addition to GA regulation in *Arabidopsis* abiotic stresses, rice regulates flooding coping submergence tolerance by two opposite GA regulations. The first quiescence strategy was that SUB1A increased the accumulation of SLENDER RICE1 (SLR1) and SLENDER RICE1 LIKE1 (SLRL1) (DELLA like proteins in rice) to restrict GA signaling and sensitivity, which resulted in inhibition of plant internode elongation and reduced carbohydrate consumption (Fukao and Bailey-Serres, 2008; Locke et al., 2018; Perata, 2018). The second deep-water escape strategy involves SK1/SK2, which lead to up-regulated *GA20oxs* and promoted internode elongation to escape submergence in water (Hattori et al., 2009; Ayano et al., 2014).

### AP2/ERFs in CTK-Mediated Stress-Response

The plant hormone CTK not only plays diverse roles in plant growth and development, but also has been reported to regulate plant abiotic stresses (Zwack and Rashotte, 2015), one of which is mediate by CRFs (Figure 3). CRFs are essential for CTK-mediated embryo, cotyledon, and leaf development, as both single and multiple CRF1/2/3/5/6 mutants displayed cell proliferation deficient phenotypes (Rashotte et al., 2006). The roles of CRF’s regulation on CTK-mediated development were further confirmed by the transcriptome analysis of *crf 1,2,5* and *crf2,3,6* mutants, with or without CTK treatment in *Arabidopsis*. About 60% of the CTK responsive genes were regulated by both CRFs and type-B ARRs (the typical cytokinin-responsive transcription factors), suggesting a model that CRFs acted tandemly with type-B ARRs to mediate CTK response. CRF6 also cooperated with CTK signaling to inhibit stress-induced leaf senescence through a common subset of CTK-regulated genes (Zwack et al., 2013). Apart from the CRFs positive effect in the CTK pathway, CRF6 also represses CTK-associated target genes involved in CTK biosynthesis, signaling and transport, to alleviate the adverse effect of CTK on abiotic stress (Zwack et al., 2016b). The opposite regulation between CRF6 and CTK on stresses and similar regulation on senescence suggest that CRF6 regulates CTK signaling through two subsets of genes: one set of genes alleviate the negative effect of CTK on abiotic stresses, while the other set helps CTK to promote plant development. The detailed mechanisms of CRF regulation in these processes remain to be determined. Identification of CRF target genes and the upstream signaling could allow for a better understanding about ERF-VIs function and how CTK regulates abiotic stresses.

### AP2/ERFs in BR-Mediated Stress-Response

The plant hormone BRs play important roles throughout plant development, such as cell elongation, leaf development, pollen tube growth, xylem differentiation, senescence, and photomorphogenesis as well as stress response (Clouse et al., 1996; Ye et al., 2017). BRs are sensed by plasma membrane located receptor kinase BRASSINOSTEROID INSENSITIVE 1 (BRI1) to inhibit negative regulator BRASSINOSTEROID INSENSITIVE 2 (BIN2), leading to accumulation of transcription factors BRASSINAZOLE-RESISTANT 2/BRI1-EMS-SUPPRESSOR 1 (BES1/BZR1) to regulate 1000s of BR responsive genes involved in plant growth and stresses responses (Guo et al., 2013).

BR regulates cold and drought responses through several pathways. For example, BR positively regulates cold tolerance partially though CBF-mediated cold response pathway, where BZR1 binds and promotes the expression of *CBF1/CBF2* in response to cold. Cold stress also promotes accumulation of the unphosphorylated and active form of BZR1 by unknown mechanisms (Li et al., 2017b; Figure 3). However, BR negatively regulates drought tolerance via antagonizing with drought induced transcription factor RD26 on drought responsive genes (Ye et al., 2017). BR also antagonizes with ABA pathway from receptors to transcription factors and regulates the trade-off of plants growth under stress conditions (Nolan T. et al., 2017). Although there are no reports of AP2/ERFs in BR mediated drought response, BES1 and BZR1 target genes include numerous AP2/ERFs, implying that AP2/ERFs have a potential function to integrate the BR pathway with abiotic stresses (Sun et al., 2010; Yu et al., 2011). Future studies in this area will shed light on the mechanisms and roles of AP2/ERFs in BR and stress responses.

In addition, AP2/ERFs regulate the BR pathway through different mechanisms. ERF72/RAP2.3 antagonizes BZR1 and AUXIN RESPONSIVE FACTOR 6 (ARF6) to inhibit hypocotyl elongation, while its role in BR regulated stresses response is unknown (Liu K. et al., 2018). The role of ERF72 in controlling growth implies that ERF72 might be a candidate for the study of the integration of BR and stresses. In fact, rice SUB1A mediates GA and BR cross-talk to control submergence tolerance. SUB1A activates BR biosynthesis and signaling, which in turn induces GA catabolic gene *GA2ox7* to lower GAs content, and ultimately promotes rice DELLA protein accumulation (Schmitz et al., 2013).

## AP2/ERF Transcription Factors and Abiotic Stresses Regulatory Network

Based on AP2/ERFs roles in abiotic stresses and hormone signaling, AP2/ERFs function through complicated regulatory networks. These networks are influenced by diverse environment stimuli and plant hormones. Regulatory mechanisms such as protein–protein interactions and cooperative or antagonistic regulation of target genes are involved in dictating the output of AP2/ERF networks for plant growth, development and abiotic stresses. The large number of the AP2/ERF transcription factors coupled with functional redundancy and their diverse roles have made it difficult to fully understand AP2/ERFs networks. A promising approach is to apply computational tools to dissect AP2/ERFs function. These include analyzing promoter sequences for abiotic stress-related motifs (Sazegari et al., 2015), and constructing stress response gene regulatory networks under different stress conditions (Dubois et al., 2017; Van den Broeck et al., 2017). For instance, analysis of 20 *Arabidopsis DREB* genes promoters demonstrated that they contained most types of HSE LTR and ABRE promoter elements. The multiple stress responsive motifs on one hand help explain the induction of *DREBs* under various environment stimuli, and on the other hand, they imply that these DREBs might form a central response network to control diverse abiotic stresses (Sazegari et al., 2015).

In addition to promoter analysis, Van den Broeck et al. examined a complex, highly interconnected network of 20 *Arabidopsis* transcription factors (more than half are AP2/ERFs) to illustrate how stress inhibits plant growth (Van den Broeck et al., 2017). Under stresses condition, plants limit growth to promote survival (Claeys et al., 2014; Bechtold and Field, 2018). In this case, members from ERF-VIIIs (ERF8, ERF9, ERF11), ERF-IXs (ERF-1, ERF2, ERF5, ERF6, ERF59, ERF98) and ERF-X (RAP2.6L) were significantly up-regulated in proliferating and expanding tissues upon short-term mannitol exposure. Among these, ERF6 (Dubois et al., 2013) and RAP2.6L (Krishnaswamy et al., 2011; Liu et al., 2012) have already been shown to positively regulate stress tolerance and inhibit growth, implying they have potential role in growth and stress trade-offs. This work also illustrated that the activation of these ERFs was sequential. ERF5, ERF6, ERF11, and ERF98 showed a fast, strong and continuous induction, while other ERFs were regulated mildly and slowly. Transcriptomic profiling of each ERF using inducible overexpression plants demonstrated that they formed a highly interconnected gene regulation network consisting of redundant regulation mediated via interaction, activation, repression, and internal regulation. By combining osmotic stress and each individual ERFs transcriptiomic data it was possible to generate a network that simplifies the signaling cascades into linear pathways, although further genetic confirmation is needed.

**FIGURE 4:**
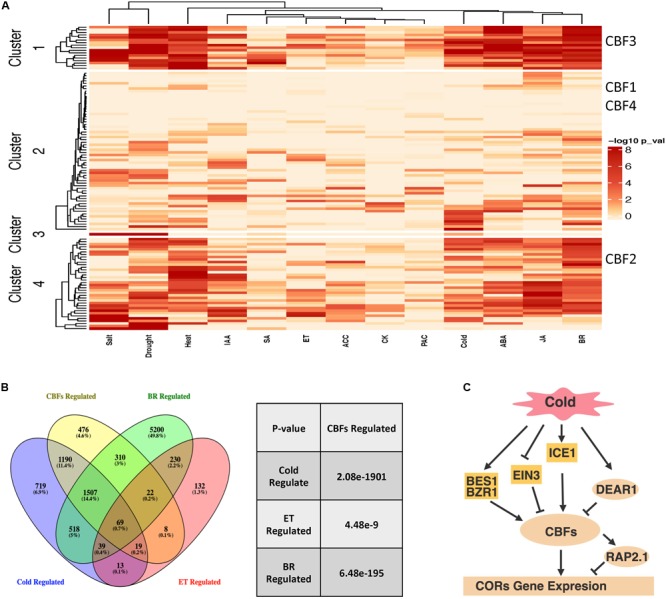
AP2/ERFs regulatory network in abiotic stresses and hormones. **(A)** *Arabidopsis* AP2/ERFs in abiotic stresses and hormone responsive gene co-expression networks. Networks from Chockalingam et al. (2016) and Chockalingam et al. (2017) were used to investigate the functions of AP2/ERFs. The color legend indicates normalized *p*-value for the enrichment of the indicated hormone- or stress-responsive gene set by Fisher’s exact test. **(B)** Venn diagram showing overlaps among CBFs-regulated genes compared with cold-regulated, ET-regulated and BR-regulated genes (left). Venny (http://bioinfogp.cnb.csic.es/tools/venny/index.html) was used to perform the comparisons. The significance of overlapping genes regulated by CBFs versus cold, ET and BR is calculated by hypergeometric test (right). **(C)** CBFs regulated cold response pathway with integration of ET and BR signaling components. Panel **(C)** is created with BioRender.

In order to gain additional insight into AP2/ERF function in abiotic stresses and hormone response pathways, we investigated *Arabidopsis AP2/ERFs* in gene co-expression networks (Chockalingam et al., 2016; Chockalingam et al., 2017) that were generated by processing and classifying 1000s of public microarray datasets into tissue and process specific categories. If AP2/ERFs are involved in hormone- and stress- responsive gene regulation, then these transcription factors would be expected to connect to hormone/stress regulated genes in the network. We used transcriptome data from various stress and hormone treatments (Maruyama et al., 2009; Bai et al., 2012; Park et al., 2015; Song et al., 2016; Hickman et al., 2017; Albihlal et al., 2018; de Zelicourt et al., 2018; Guo et al., 2018; Xie et al., 2018; Zhang F. et al., 2018) to test this idea and found that many AP2/ERFs connect to more hormone/stress regulated genes in the network than would be expected by chance (Figure 4A, Fisher’s exact test). We further divided AP2/ERFs into four clusters based on enrichment for hormone or stress responsive genes in the network. The role of CBFs in stress and hormone responses was investigated as an example. CBFs are distributed into four clusters that include AP2/ERFs enriched for cold, drought, salt and heat responsive genes. These clusters also include AP2/ERFs enriched for ABA, ET, and BR responsive genes. In line with these network predictions, the function of CBFs in ET and cold stress (including those mediated by BRs) has been confirmed by genetics or transcriptomic studies (Shi et al., 2012; Li et al., 2017b). We further compared genes differentially expressed in *cbf123* triple mutants after cold treatment, BR and ET responsive genes (Figure 4B; Zhao et al., 2016; Nolan T. M. et al., 2017; de Zelicourt et al., 2018; Zhang F. et al., 2018). The comparison indicates that CBFs share a significant amount of genes with cold, ET and BR. For example, cold regulates ∼4000 genes, ∼68% of which are regulated by CBFs, supporting CBFs’ major contribution in cold tolerance. Among these, ∼20% of CBF regulated cold responsive genes are regulated by ET and BR, indicating that ET and BR regulated cold stress are dependent on the CBF pathway. ET and BR negatively and positively regulated CBFs through their specific transcription factors EIN3 (Shi et al., 2012) and BES1/BZR1 (Li et al., 2017b), respectively (Figure 4C). Taken together, these examples of network and transcriptome comparisons can (1) predict AP2/ERFs functions, (2) study AP2/ERFs function in specific stress/hormone responses; (3) examine the cross-talk of hormones in controlling growth and stress tolerance; (4) help clarify their upstream regulators and downstream targets.

## Summary and Future Directions

In summary, AP2/ERFs are regulated by stresses and control multiple plant stress responses to coordinate plant growth under stress conditions. AP2/ERFs are both regulated by plant hormone pathways and modulate plant hormone biosynthesis and signaling. Considering the complex roles of AP2/ERFs in abiotic stress and hormonal responses, future studies are required to fully understand this unique family of transcription factors. The regulation of AP2/ERFs by various hormone signaling pathways should be examined at transcriptional and post-transcriptional levels. The genome-wide identification of AP2/ERF target genes should help understand their functions as well as gauge the scope of their actions. Establishing gene regulatory networks using gene expression data and computational tools will be crucial to understand the large family of genes with 100s–1000s of target genes. Considering the complexity, the functions of AP2/ERFs should be examined in different tissues/cell types and in a temporal manner. Many AP2/ERFs are potential candidates for crops stress tolerance engineering. Thus, a full understanding of AP2/ERFs can guide crop engineering to achieve optimal plant growth and crop production under changing environmental conditions.

## Data Availability

Publicly available datasets were analyzed in this study. This data can be found here: http://www.plantphysiol.org/content/171/4/2744/tab-figures-data.

## Author Contributions

ZX, TN, HJ, and YY conceived the topic. TN and HJ collected the gene regulation data. TN performed the network analysis. ZX wrote the manuscript with edits from other co-authors.

## Conflict of Interest Statement

The authors declare that the research was conducted in the absence of any commercial or financial relationships that could be construed as a potential conflict of interest.
